# Long-Term Outcomes After In-Hospital Cardiac Arrest: Does Pre-arrest Skeletal Muscle Depletion Matter?

**DOI:** 10.3389/fphys.2021.692757

**Published:** 2021-07-29

**Authors:** Seok-In Hong, Kyung Won Kim, Yousun Ko, Youn-Jung Kim, Jin Won Huh, Sang-Bum Hong, Won Young Kim

**Affiliations:** ^1^Department of Emergency Medicine, University of Ulsan College of Medicine, Asan Medical Center, Seoul, South Korea; ^2^Department of Radiology, University of Ulsan College of Medicine, Asan Medical Center, Seoul, South Korea; ^3^Biomedical Research Center, Asan Institute for Life Sciences, Asan Medical Center, Seoul, South Korea; ^4^Department of Pulmonary and Critical Care Medicine, University of Ulsan College of Medicine, Asan Medical Center, Seoul, South Korea

**Keywords:** in-hospital cardiac arrest, long-term outcome, neurologic outcome, sarcopenia, skeletal muscle depletion

## Abstract

**Background:** Skeletal muscle depletion is prevalent in elderly patients and is associated with unfavorable outcomes in patients with chronic diseases. However, the relationship between skeletal muscle mass and neurological outcomes following in-hospital cardiac arrest (IHCA) has not been evaluated. The aim of this study was to investigate whether skeletal muscle status before cardiac arrest is an independent factor affecting neurological outcomes in patients with IHCA.

**Methods:** We reviewed a prospectively enrolled registry of IHCA patients. Consecutive adult patients (>18 years) admitted to a tertiary care hospital from 2013 to 2019 were included in the study. Of these, 421 patients who underwent abdominopelvic computed tomography within 3 months of cardiac arrest were included. Skeletal muscle index (SMI) was measured at the third lumbar vertebra, and skeletal muscle depletion was defined using sex- and body mass index-specific cutoffs of SMI. The primary outcome was a Cerebral Performance Category score of 1 or 2 at 6 months after cardiac arrest, which was considered a good neurological outcome.

**Results:** Of the 421 patients, 248 (58.9%) had skeletal muscle depletion before IHCA. The patients without skeletal muscle depletion showed significantly better neurological outcomes at 6 months after cardiac arrest than those with pre-arrest muscle depletion (20.8 vs. 10.9%, *p* = 0.004). The absence of skeletal muscle depletion was significantly associated with good neurological outcomes in a multivariable logistic analysis (OR = 3.49, 95% confidence intervals: 1.83–6.65, *p* < 0.001), along with the absence of diabetes, presence of active cancer, shockable rhythm, and short resuscitation duration.

**Conclusion:** Pre-arrest skeletal muscle depletion was associated with long-term mortality and poor neurological outcomes after IHCA. Skeletal muscle depletion may be used as a tool to identify at-risk patients who may benefit from aggressive treatments.

## Introduction

The incidence of in-hospital cardiac arrest (IHCA) has increased to nearly 1 out of every 339 hospitalized adults (Kazaure et al., 2013). Although advances in cardiopulmonary resuscitation (CPR) have increased the survival rate to 22.3% (Girotra et al., 2012), the likelihood of a good outcome after CPR, defined as a Cerebral Performance Category (CPC) score of 1 or 2 (Rittenberger et al., 2011), remains poor following IHCA. The objective prediction of good outcomes for IHCA before cardiac arrest can inform treatment decisions and provide adequate information to family members (Yoon et al., 2018; Kang et al., 2019; George et al., 2020).

Loss of muscle mass and function, known as “sarcopenia,” is prevalent in the elderly (Marzetti et al., 2017). Muscle depletion is related to cardiac function and is a predictor of unfavorable outcomes in patients with chronic diseases (Martin et al., 2013; Moisey et al., 2013; Wong and Frishman, 2019). However, the predictive value of skeletal muscle status for good neurological outcomes after IHCA has not been investigated. Several methods are available to quantify skeletal muscle mass, including dual-energy X-ray absorptiometry (DXA) and bioelectrical impedance analysis (BIA) (Cruz-Jentoft et al., 2019). Measurement of skeletal muscle mass by computed tomography (CT), most commonly obtained at the third lumbar vertebra, is a widely described alternative method for measuring skeletal muscle mass (Mourtzakis et al., 2008).

Obesity despite skeletal muscle depletion is a medical condition defined as the coexistence of sarcopenia and obesity (Polyzos and Margioris, 2018). The health-related risks associated with sarcopenic obesity may be equal to or greater than the sum of the respective risks of obesity and sarcopenia alone. However, the potential synergy of obesity and sarcopenia as a syndromic entity remains hypothetical. Moreover, the relationship between sarcopenic obesity and outcomes in patients with IHCA has not been determined yet.

We hypothesize that the long-term prognosis of patients with IHCA is associated with pre-arrest skeletal muscle depletion. Thus, the aim of this study was to investigate whether pre-arrest muscle mass status, such as the presence of skeletal muscle depletion, is an independent factor associated with a higher likelihood of poor neurological outcomes after IHCA.

## Materials and Methods

### Study Setting and Patient Population

This retrospective cohort study was conducted at Asan Medical Center, a 2700-bed tertiary care hospital in Seoul, Korea. We used a prospectively enrolled registry of IHCA patients to enroll consecutive adult patients (>18 years) admitted to the hospital from March 2013 to February 2019. We identified patients who underwent abdominopelvic computed tomography (APCT) within 3 months before cardiac arrest. The IHCA patients who already had poor cerebral performance (CPC score 3–5) before cardiac arrest and those with inadequate APCT images, no height data, and/or no recorded outcomes were excluded. The ethics committee of Asan Medical Center approved the study protocols and waived the need for informed consent. Personal information of all patients was anonymized and removed before analysis.

### CPR Team and IHCA Registry

The medical emergency team (MET) system has been employed at Asan Medical Center since March 2008 (Huh et al., 2014). Physicians and nurses involved in the team are responsible for the identification and critical care of patients who have a risk of cardiac arrest. As members of the cardiac arrest team, the MET manages all IHCA patients even after CPR. After CPR, nurses fill in the CPR registry online within 24 h. The data in the IHCA registry are reviewed and validated by the MET and resuscitation committee. The registry data include demographic history (e.g., age, sex, medical history, diagnosis at hospital visits), resuscitation profiles (e.g., location, initial cardiac rhythm when cardiac arrest was identified), critical interventions conducted at the time of cardiac arrest, defibrillation during resuscitation, time required to defibrillate, and outcomes (e.g., survival, neurological status).

A CPC score was used to determine the neurological status after cardiac arrest (Yoon et al., 2018). For CPC scoring, patients were classified into five categories (Ohlsson et al., 2016): 1 (good cerebral performance: conscious, able to work, might have mild neurological or psychological deficit); 2 (moderate cerebral disability: conscious, sufficient cerebral function for independent activities of daily life); 3 (severe cerebral disability: conscious, dependent on others for daily support because of impaired brain function); 4 (coma or vegetative state: any degree of coma without the presence of any brain death criteria); and 5 (brain death).

### Data Collection

**Figure 1:**
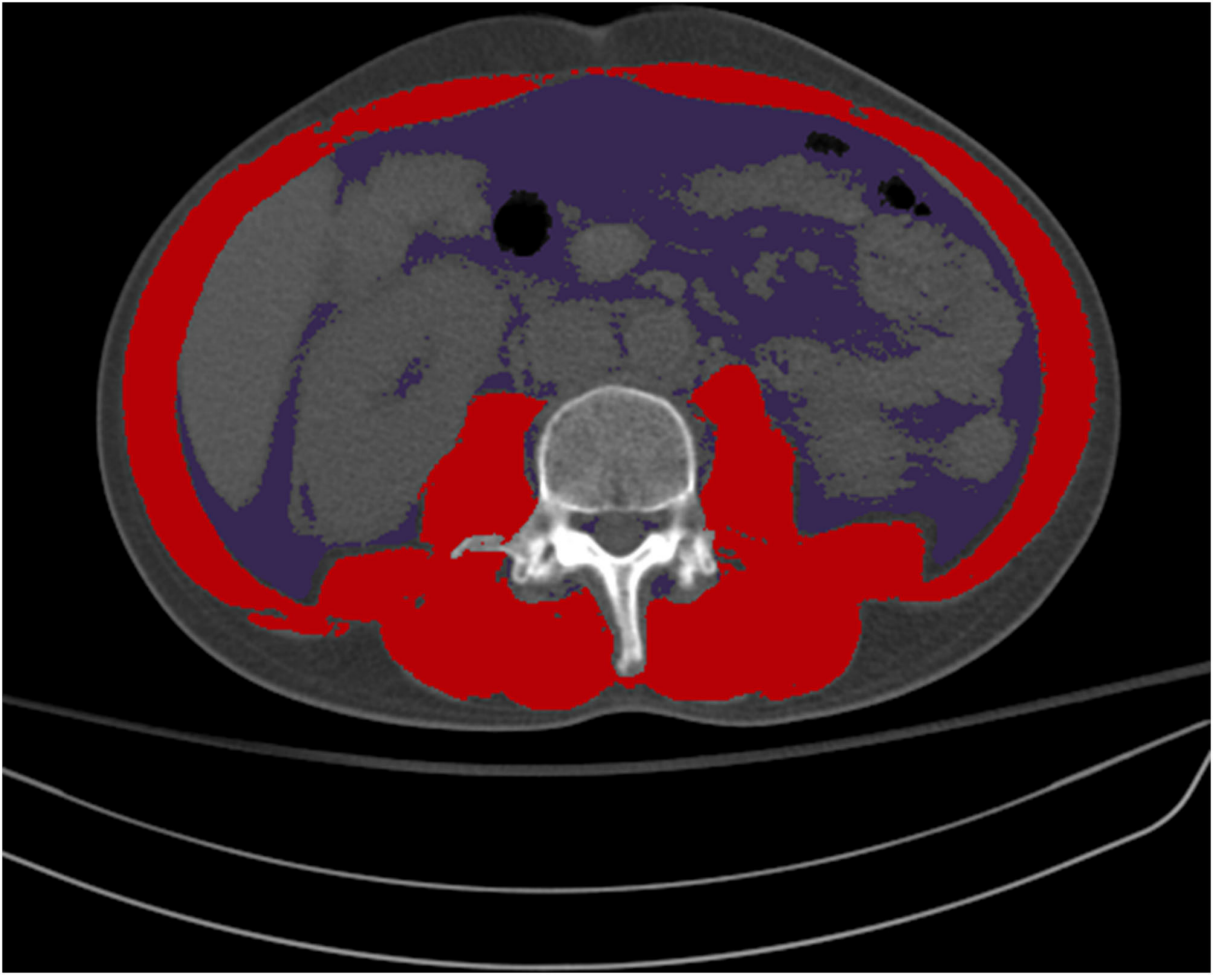
Color demarcation for skeletal muscle area (red) and visceral fat area (purple) at the third lumbar vertebra.

Survival rates and neurological outcomes at discharge and at 1 and 6 months after IHCA were retrieved from the registry. Additional data were obtained using the electronic medical records, including weight, height, and the presence of an APCT scan to evaluate body composition. We retrieved body weight and height from the electronic health records on the day closest to when the CT scan was performed. Skeletal muscle area (SMA; cm^2^) and visceral fat area (VFA; cm^2^) were assessed at the third lumbar vertebra (L3) of the APCT scan. APCT images extending from L3 in the inferior direction were assessed. Body composition on CT was evaluated using an artificial intelligence software (AID-U™, iAID inc. Seoul, Korea) developed using a fully convolutional network segmentation technique (Park et al., 2020). Two experienced operators (Y. K and K. W. K), who were blind to clinical information, selected axial CT slices at the L3 inferior endplate level in a semi-automatic manner using coronal reconstructed images. Selected CT images were automatically segmented to generate the boundary of total abdominal muscles and measure the abdominal muscle and fat area (Figure 1). Next, two operators (Y. K and K. W. K) checked the quality of the muscle segmentation in all images. The SMA was demarcated using predetermined thresholds [−29 to +190 Hounsfield units (HU)], and the VFA was demarcated using fat tissue thresholds (−190 to +30 HU). Skeletal muscle attenuation was assessed as the mean radiodensity in HU of all SMA at L3.

### Definitions

The body mass index (BMI) was calculated as weight in kg divided by height in meters squared (kg/m^2^). BMI values were categorized as underweight (<18.5 kg/m^2^), normal (18.5–22.9 kg/m^2^), overweight (23.0–24.9 kg/m^2^), or obese (≥25.0 kg/m^2^), using cutoffs appropriate for the Asian population (WHO Expert Consultation, 2004).

The skeletal muscle index (SMI) was calculated as the SMA in cm squared (measured at L3) divided by the height in meters squared (cm^2^/m^2^). Skeletal muscle depletion was defined as an SMI of <43 in men with a BMI of <25, <53 in men with a BMI of ≥25, or <41 in women regardless of the BMI. The sex- and BMI-specific cutoff values were based on survival data of patients with cancer (Martin et al., 2013). A linear relation between the L3 muscle area measured by CT and the appendicular skeletal muscle (kg/m^2^) measured by DXA was reported (Mourtzakis et al., 2008). Skeletal muscle depletion with obesity was defined as a depletion of skeletal muscle in patients with BMI of ≥25 regardless of sex (Kamo et al., 2019).

Patients who had tumor burden or received chemo- or radiotherapy within 3 months of cardiac arrest were classified as having active cancer.

### Statistical Analysis

Data are presented as numbers and percentages for categorical variables. Continuous variables are described using medians with interquartile ranges (IQR) because they were not normally distributed. Differences between groups were analyzed using Chi-square and Fisher's exact test for categorical variables and Mann–Whitney U test for continuous variables, as appropriate. The relationship between skeletal muscle depletion and neurological outcomes was examined using univariable and multivariable logistic regression analyses. The results are presented as crude odds ratios (OR) for univariable analysis and adjusted OR for multivariable analysis, with 95% confidence intervals (CI). Variables with *P*-values of <0.1 in univariable analysis were selected for multivariable analysis. The purposeful selection of those variables using the standard *p*-value of <0.1 was based on the recommendations from previous studies (Bursac et al., 2008; Wells et al., 2012). Considering the number of variables, a backward elimination method for stepwise regression analysis was performed to compare with other well-known factors predicting the outcomes of cardiac arrest. The variables included in the multivariable logistic regression analysis were tested for multicollinearity by calculating Pearson's correlation coefficients and variance inflation factors. A variable with a coefficient of <0.7 and variance inflation factor of <10.0 was considered to have no multicollinearity with other variables. Two-tailed *P*-values of <0.05 were considered statistically significant. All statistical analyses were performed using IBM SPSS Statistics for Windows, version 21.0 (IBM Corp., Armonk, NY, USA).

## Results

### Patient Population

**Figure 2:**
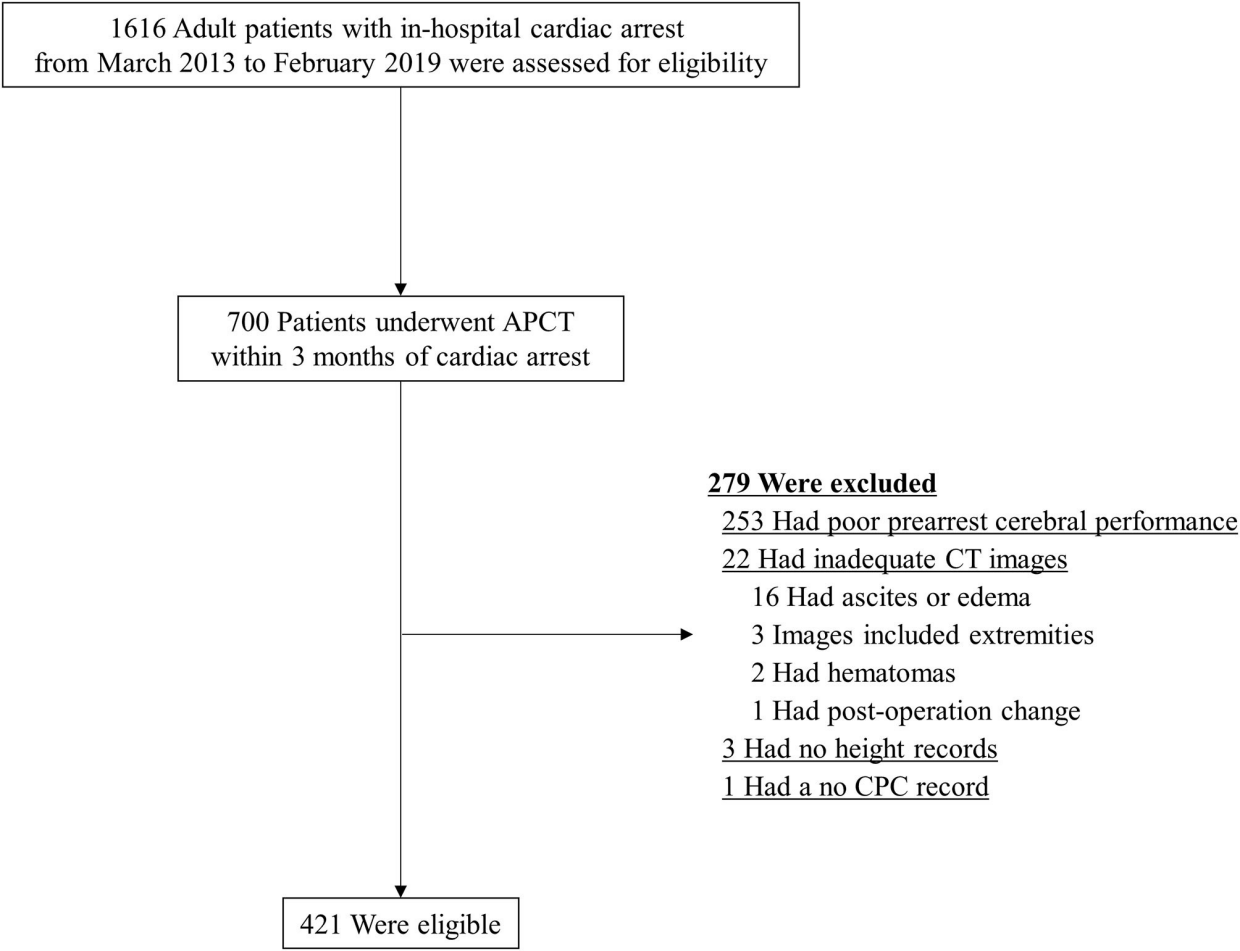
Patient flow diagram. APCT, abdominopelvic computed tomography; CPC, Cerebral Performance Score; CT, computed tomography.

Of the 1,616 patients with IHCA, 700 patients underwent APCT within 3 months before cardiac arrest, of whom 279 were excluded. Reasons for exclusion included poor cerebral performance (*n* = 253) and inadequate CT image for the evaluation of SMI or lacking an essential value in the electronic medical records (*n* = 26), as shown in Figure 2. The remaining 421 patients were included in this study.

Baseline clinical data are presented in Table 1. Patients were categorized into skeletal muscle depletion and non-skeletal muscle depletion groups. Except for demographics, no significant differences in comorbidities, diagnosis at admission, arrest characteristics, and duration from the day of APCT to IHCA were detected between the two groups. The median age of patients with skeletal muscle depletion was 67.0 years (IQR, 56.0–76.0), which was significantly higher than the median age of 63.0 years (IQR, 54.0–72.0; *p* = 0.019) for patients without depletion. The proportion of males was significantly lower in the skeletal muscle depletion group than in the non-skeletal muscle depletion group (54.8 vs. 68.8%, respectively; *p* = 0.005). In patients with skeletal muscle depletion, the median BMI (21.8 vs. 23.9 kg/m^2^; *p* < 0.001) and SMI (37.2 vs. 48.3 cm^2^/m^2^; *p* < 0.001) were significantly lower than in patients without depletion because BMI-specific cutoff values were used to diagnose skeletal muscle depletion. Median VFA was also significantly lower in patients with skeletal muscle depletion than in those without depletion (94.5 vs. 115.3 cm^2^; *p* = 0.007).

**Table 1:** Clinical characteristics of patients with in-hospital cardiac arrest included in the study.

**Characteristics** ^†^	**All patients**	**Non-skeletal muscle depletion**	**Skeletal muscle depletion**	***p*** ^*^
	**(*N* = 421)**	**(*N* = 173)**	**(*N* = 248)**	
Demographics				
Age (years)	65.0 (55.0–74.0)	63.0 (54.0–72.0)	67.0 (56.0–76.0)	0.019
Male	255 (60.6)	119 (68.8)	136 (54.8)	0.005
Comorbidities				
Hypertension	162 (38.5)	65 (37.6)	97 (39.1)	0.761
Diabetes mellitus	136 (32.3)	56 (32.4)	80 (32.3)	1.000
Coronary artery disease	62 (14.7)	27 (15.6)	35 (14.1)	0.677
Heart failure	53 (12.6)	21 (12.1)	32 (12.9)	0.882
Chronic pulmonary disease	28 (6.7)	14 (8.1)	14 (5.6)	0.328
Chronic kidney disease	73 (17.3)	31 (17.9)	42 (16.9)	0.795
Liver cirrhosis	52 (12.4)	20 (11.6)	32 (12.9)	0.764
Active cancer	224 (53.2)	98 (56.6)	126 (50.8)	0.275
Diagnosis at admission				
Cardiac	72 (17.1)	30 (17.3)	42 (16.9)	1.000
Other medical	278 (66.0)	113 (65.3)	165 (66.5)	0.835
Surgical	70 (16.6)	29 (16.8)	41 (16.5)	1.000
Characteristics of arrest				
Witnessed	370 (87.9)	148 (85.5)	222 (89.5)	0.228
Shockable rhythm	69 (16.4)	24 (13.9)	45 (18.1)	0.285
Resuscitation duration (min)	11.0 (4.0–25.0)	11.0 (4.0–27.0)	11.0 (4.0–24.0)	0.700
Presumed cardiac cause	81 (19.2)	34 (19.7)	47 (19.0)	0.900
CASPRI score^a^	20.0 (16.0−24.0)	20.0 (16.0–23.0)	20.0 (17.0–25.0)	0.184
GO-FAR score^b^	7.0 (0.0–13.0)	6.0 (0.0–13.0)	7.0 (0.0–14.0)	0.380
Physical characteristics				
Body mass index (kg/m^2^)	22.9 (19.8–25.3)	23.9 (21.9–25.9)	21.8 (19.1–25.0)	<0.001
Underweight (<18.5)	48 (11.4)	4 (2.3)	44 (17.7)	
Normal (18.5–22.9)	167 (39.7)	61 (35.3)	106 (42.7)	
Overweight (23.0–24.9)	84 (20.0)	48 (27.7)	36 (14.5)	
Obese (≥25.0)	122 (29.0)	60 (34.7)	62 (25.0)	
SMI (cm^2^/m^2^)	41.3 (36.2–48.0)	48.3 (45.2–54.0)	37.2 (33.7–40.0)	<0.001
VFA (cm^2^)	103.9 (55.7–155.9)	115.3 (70.9–156.4)	94.5 (48.4–153.1)	0.007
APCT to IHCA (days)	11.0 (2.0–31.0)	13.0 (2.0–36.0)	10.0 (2.0–27.0)	0.152

*APCT, abdominopelvic computed tomography; CASPRI, cardiac arrest survival post-resuscitation In-hospital; CPC, cerebral performance category; GO-FAR, good outcome following attempted resuscitation; IHCA, in-hospital cardiac arrest; ROSC, return of spontaneous circulation; SMI, skeletal muscle index; VFA, visceral fat area*.

† *Continuous variables are expressed as median with interquartile ranges; categorical values are expressed as a number with a percentage*.

* *p-value was calculated using Chi-square and Fisher's exact tests (categorical variables) and Mann–Whitney U test (continuous variables) where appropriate*.

a,b *CASPRI and GO-FAR score are useful tools for predicting neurological outcome following IHCA, and were validated in an Asian population (Wang et al., 2018; Cho et al., 2020)*.

### Outcomes

The overall survival rate 6 months after IHCA was 19.0%, with 15.0% of all patients having a good neurological outcome, as defined by a CPC score of 1 or 2. Our study revealed that the depletion of skeletal muscle was significantly related to long-term mortality and poor neurological outcomes of patients after IHCA. Significantly more patients without skeletal muscle depletion had CPC scores of 1–2 at the time of discharge from the hospital (26.6 vs. 12.1%; *p* < 0.001) and at 1 month (26.6 vs. 11.3%; *p* < 0.001) and 6 months (20.8 vs. 10.9%; *p* = 0.004) after IHCA compared with patients with skeletal muscle depletion, as shown in Table 2. However, no significant differences in sustained return of spontaneous circulation were detected.

**Table 2:** Neurological outcomes and survival rates of patients in relation to skeletal muscle depletion.

**Outcomes**	**All patients (*N* = 421)**	**Non-skeletal muscle depletion(*N* = 173)**	**Skeletal muscle depletion (*N* = 248)**	***p*** ^*^
Sustained ROSC	292 (69.4)	124 (71.7)	168 (67.7)	0.452
At discharge				
Survival	112 (26.6)	56 (32.4)	56 (22.6)	0.033
Good (CPC 1–2)	76 (18.1)	46 (26.6)	30 (12.1)	<0.001
At 1 month				
Survival	103 (24.5)	53 (30.6)	50 (20.2)	0.016
Good (CPC 1–2)	74 (17.6)	46 (26.6)	28 (11.3)	<0.001
At 6 months				
Survival	80 (19.0)	42 (24.3)	38 (15.3)	0.015
Good (CPC 1–2)	63 (15.0)	36 (20.8)	27 (10.9)	0.004

*CPC, Cerebral Performance Score; ROSC, return of spontaneous circulation*.

*Data are expressed as a number with a percentage*.

* *p-value was calculated by using Chi-square and Fisher's exact test for categorical variables*.

Variables were also examined by univariable logistic regression analysis and those with *p*-values of <0.1 were entered into a multivariable model (Supplementary Table 1). There was no multicollinearity among the variables. The absence of skeletal muscle depletion was significantly associated with good neurological outcomes, according to the results of the multivariable logistic regression analysis (OR = 3.489, 95% CI: 1.830–6.651; *p* < 0.001), along with the presence of shockable rhythm (OR = 2.330, 95% CI: 1.066–5.093; *p* = 0.034). Good neurological outcomes were inversely related to other variables, including the presence of diabetes mellitus (OR = 0.412, 95% CI: 0.197–0.859; *p* = 0.018) and the presence of active cancer (OR = 0.402, 95% CI: 0.203–0.797; *p* = 0.009). The longer the resuscitation duration, the less likely the patients were to achieve a good outcome (OR = 0.909, 95% CI: 0.873–0.946; *p* < 0.001). The other two variables (witnessed and presumed cardiac cause) did not reach statistical significance (Table 3).

**Table 3:** Multivariable logistic analysis for good neurological outcome at 6 months.

**Variables** ^†^	**Good neurological outcome at 6 months**	
	**Adjusted OR (95% CI)**	***p*** *
Presence of diabetes mellitus	0.412 (0.197–0.859)	0.018
Presence of active cancer	0.402 (0.203–0.797)	0.009
Witnessed	7.546 (0.977–58.308)	0.053
Presence of shockable rhythm	2.330 (1.066–5.093)	0.034
Resuscitation duration	0.909 (0.873–0.946)	<0.001
Presumed cardiac cause	2.129 (0.962–4.713)	0.062
Absence of skeletal muscle depletion	3.489 (1.830–6.651)	<0.001

*CI, confidence interval; CPC, cerebral performance score; OR, odds ratio*.

† *Variables with p-values of <0.1 in univariable analysis were entered into a multivariable analysis*.

* *p-value was calculated from the multivariable logistic regression analysis after using backward elimination methods*.

A subgroup analysis was conducted in patients with obesity having a BMI of ≥25. Neurological outcomes were compared among patients with skeletal muscle depletion according to the presence of obesity. The rates of survival and good neurological outcome were higher in patients without obesity than in those with obesity at the time of discharge and at 1 and 6 months after arrest. However, these relationships were not statistically significant (Table 4).

**Table 4:** Relationship between skeletal muscle depletion and good neurological outcome according to the presence of obesity.

	**Skeletal muscle depletion (** ***N*** **=** **248)**		
**Outcomes**	**Non-obese, BMI** ^†^ **<25**	**Obese, BMI ≥ 25**	***p*** ^*^
	**(*N* = 186)**	**(*N* = 62)**	
Sustained ROSC	129 (69.4)	39 (62.9)	0.351
At discharge			
Survival	45 (24.2)	11 (17.7)	0.381
Good (CPC 1–2)	22 (11.8)	8 (12.9)	0.824
At 1 month			
Survival	37 (19.9)	13 (21.0)	0.856
Good (CPC 1–2)	20 (10.8)	8 (12.9)	0.646
At 6 months			
Survival	28 (15.1)	10 (16.1)	0.840
Good (CPC 1–2)	19 (10.2)	8 (12.9)	0.638

*BMI, body mass index; CPC, cerebral performance category; ROSC, return of spontaneous circulation. Data are expressed as a number with a percentage*.

† *Obesity is defined as BMI of ≥25 kg/m^2^, and the units are omitted in the table*.

* *p-value was calculated using Chi-square and Fisher's exact test for categorical variables*.

## Discussion

The European Working Group on Sarcopenia in Older People updated the definition of sarcopenia in 2018 (Cruz-Jentoft et al., 2019) and reported that sarcopenia is associated with cardiac disease (Bahat and Ilhan, 2016), respiratory disease (Bone et al., 2017), cognitive impairment (Chang et al., 2016), need for long-term care placement (Steffl et al., 2017), and death (De Buyser et al., 2016). Sarcopenia has been studied in various medical areas to identify links between muscle pathology and adverse health outcomes. The principal finding of this study is that patients with no pre-arrest depletion of skeletal muscle have a good prognosis after IHCA. Skeletal muscle is depleted owing to aging, development of chronic diseases, physical inactivity, and inappropriate nutrition (Cruz-Jentoft et al., 2019). Therefore, muscle mass reflects patients' pre-disease performance status, which affects outcomes after cardiac arrest. A possible explanation for these findings is that skeletal muscle is profoundly related to the inflammatory process and acts as an endocrine organ. Skeletal muscle secretes cytokines and other hormone-like factors, called myokines, which counteract the harmful effects of the proinflammatory cytokines (Pedersen, 2009). Dysfunctional myokine secretion may contribute to unfavorable outcomes in patients with IHCA (Walsh, 2009). Based on the results of this study, long-term neurological outcomes can be more accurately predicted by identifying skeletal muscle status along with other well-known factors influencing outcomes after IHCA (Wang et al., 2018; Cho et al., 2020). The presence of a shockable rhythm and short resuscitation duration were associated with good neurological outcomes in our study, as previously published (Andersen et al., 2019). Furthermore, preexisting medical conditions such as diabetes and malignancy are strongly associated with outcomes following IHCA (Ebell and Afonso, 2011).

Despite the association between skeletal muscle depletion and 6-month neurological outcomes, neurological prognostication by skeletal muscle depletion after the sixth month of cardiac arrest cannot be guaranteed. Skeletal muscle quantity can be altered over time in response to the external environment or disease progression, unlike other factors (e.g., diabetes, hepatic disease, history of coronary arterial disease) that influence patient outcomes. For example, a patient who does not have skeletal muscle depletion may develop muscle depletion within a few months as cancer progresses, and vice versa. Alterations in muscle quantity after cardiac arrest should be considered when explaining the association between pre-arrest muscle status and neurological outcomes at 6 months after IHCA. Conversely, interventions that can reverse or prevent skeletal muscle depletion have not been identified. Moreover, the outcomes of patients who overcome muscle depletion status are unknown. This area requires further research.

Sarcopenic obesity results in poor outcomes in various diseases and operations (Montano-Loza et al., 2016; Zhang et al., 2019). However, when we defined obesity as a BMI of ≥25, skeletal muscle depletion and neurological outcomes were not significantly related in patients with obesity. Patients with obesity tended to have poorer outcomes than non-obese patients, but the difference was not statistically significant. Likewise, patients who had VFA-defined obesity and skeletal muscle depletion were more prone to have poor neurological outcomes; however, no significant difference was found compared with patients with only skeletal muscle depletion (Supplementary Table 2). BMI may not be accurate in patients with ascites, pulmonary or soft-tissue edema, and other conditions that affect fluid balance. The VFA could reflect the subject's obesity more precisely even in the presence of ascites or edema, but the statistical power was weak. To validate relationships among fat quantities, skeletal muscle depletion, and outcomes of cardiac arrests, future research is warranted.

One limitation of this study was the evaluation of skeletal muscle status by CT-defined muscle quantities. The term “sarcopenia” means loss of skeletal muscle and its function; therefore, we use the term “skeletal muscle depletion” rather than sarcopenia. Muscle function can be measured by various tests, including the grip strength test (Cruz-Jentoft et al., 2019). However, muscle function was not routinely measured in our hospital during the time the study population was admitted. Including muscle strength measurement in future investigations may improve the predictive ability of sarcopenia in relation to neurological outcomes. Recently, efforts were made to find new biomarkers to diagnose sarcopenia and loss of muscle mass (Drescher et al., 2015). Identification of such new diagnostic tools will improve and build upon our results when applied to medical practice.

Compared with other methods for skeletal muscle quantity measurement, such as DXA and BIA, CT exposes the patients to radiation. Therefore, we retrieved SMI from CT scans that were acquired for diagnostic purposes. As such, we only included patients who underwent APCT and excluded patients without diseases originating from the abdomen or pelvic cavities; moreover, patients with IHCA have several comorbidities, thereby adding to a possible selection bias in this study. Patients with active cancer accounted for more than half of the total study population (53.2%), which would have served as another selection bias as well.

**Figure 3:**
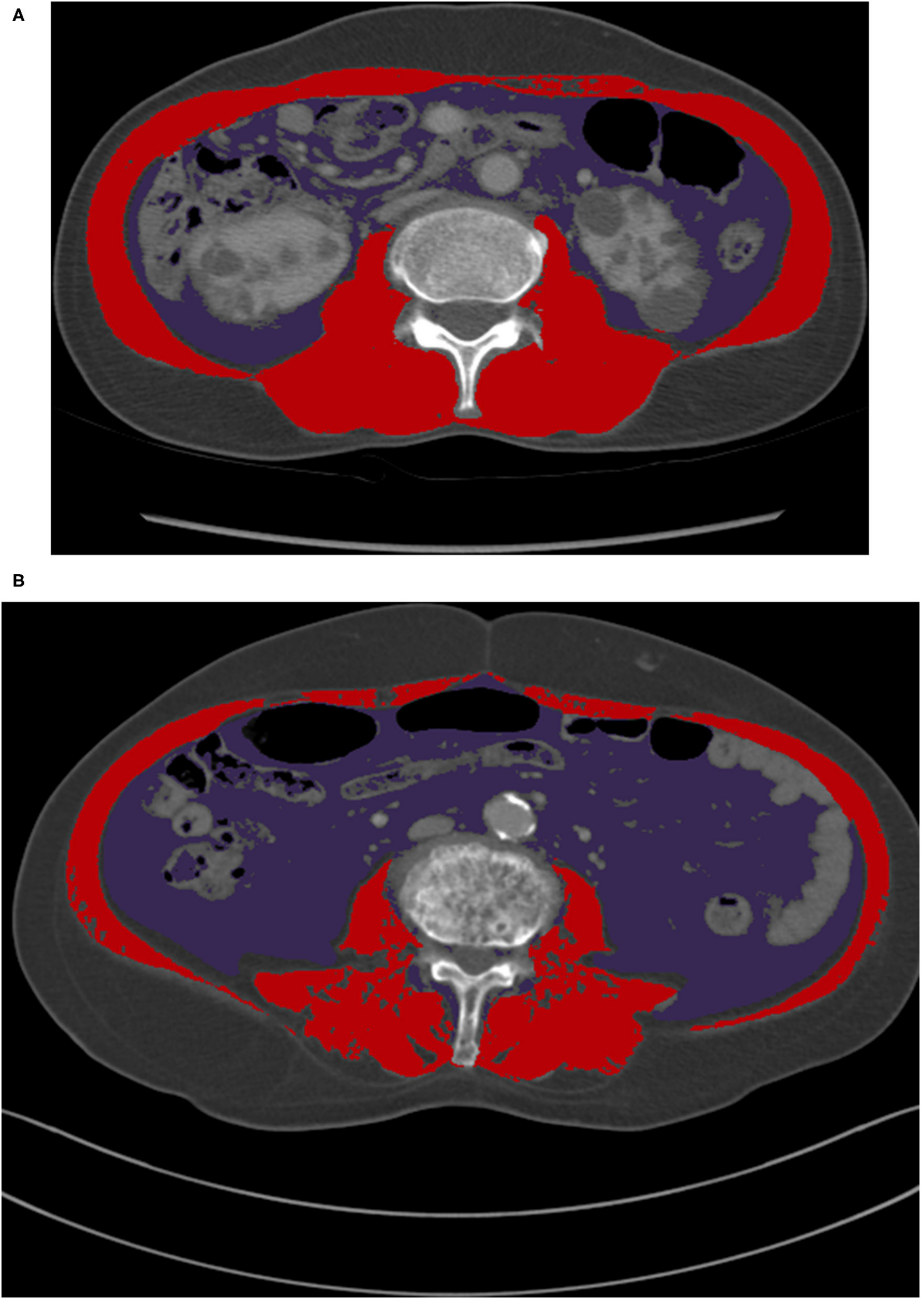
A comparison of CT images at L3 for male patients with normal BMI categories. **(A)** A patient who had skeletal muscle depletion only (SMI of 38.3 cm^2^/m^2^ and VFA of 61.5 cm^2^), **(B)** A patient who had visceral fat obesity and skeletal muscle depletion (SMI of 35.9 cm^2^/m^2^ and VFA of 100.1 cm^2^). BMI, body mass index; CT, computed tomography; L3, the third lumbar vertebra; SMI, skeletal muscle index; VFA, visceral fat area.

Despite these limitations, our study results may be applied in in-patients with CT scans, particularly in the area of critical care, because the data were derived from APCT scans. A diagnostic APCT scan to evaluate various diseases is widely used and its use is rapidly increasing (Levin et al., 2017). A physician could assess the skeletal muscle status of at-risk patients with severe illness by identifying previously obtained CT images. Muscle depletion status can be used as a tool in conjunction with other existing prediction models (i.e., GO-FAR score) to identify patients who may benefit from aggressive treatments and to make decisions regarding do-not-resuscitation orders before cardiac arrest. Moreover, simple visualization of the typical presentation of skeletal muscle depletion can be achieved by physicians with CT scan images, although specific techniques and experienced operators are needed to accurately calculate the muscle mass (Figure 3).

Although it is a single-centered observational study, our research included a relatively large number of patients. However, the study population does not represent the characteristics of the general population owing to previously mentioned possible selection biases. Therefore, external validation of our study results is needed.

## Conclusion

Pre-arrest skeletal muscle depletion was associated with long-term mortality and neurologically poor outcomes at 6 months after IHCA. However, skeletal muscle depletion with obesity did not affect the relationship between skeletal muscle depletion and neurological outcomes.

## Data Availability Statement

The raw data supporting the conclusions of this article will be made available by the authors, without undue reservation.

## Ethics Statement

The studies involving human participants were reviewed and approved by the ethics committee of Asan Medical Center. Written informed consent for participation was not required for this study in accordance with the national legislation and the institutional requirements.

## Author Contributions

S-IH, KK, YK, Y-JK, and JH: acquisitions of data. S-IH: drafting of the manuscript. KK, S-BH, and WK: critical revision of manuscript. S-IH and KK: statistical analysis. All authors study concept and design, read and approved the final manuscript.

## Conflict of Interest

KK is an inventor of a patent issued by the Korean Intellectual Property Office (KR patent application No. 10-2018-0035284). It is an image processing method for adopting human body morphometric based on artificial neural network for sarcopenia. The remaining authors declare that the research was conducted in the absence of any commercial or financial relationships that could be construed as a potential conflict of interest.

## Publisher's Note

All claims expressed in this article are solely those of the authors and do not necessarily represent those of their affiliated organizations, or those of the publisher, the editors and the reviewers. Any product that may be evaluated in this article, or claim that may be made by its manufacturer, is not guaranteed or endorsed by the publisher.

## References

[B1] Andersen L. W. Holmberg M. J. Berg K. M. Donnino M. W. Granfeldt A. (2019). In-hospital cardiac arrest: a review. JAMA 321, 1200–1210. 10.1001/jama.2019.1696 30912843 PMC6482460

[B2] Bahat G. Ilhan B. (2016). Sarcopenia and the cardiometabolic syndrome: a narrative review. Eur. Geriatr. Med. 7, 220–223. 10.1016/j.eurger.2015.12.012

[B3] Bone A. E. Hepgul N. Kon S. Maddocks M. (2017). Sarcopenia and frailty in chronic respiratory disease. Chron. Respir. Dis. 14, 85–99. 10.1177/1479972316679664 27923981 PMC5720213

[B4] Bursac Z. Gauss C. H. Williams D. K. Hosmer D. W. (2008). Purposeful selection of variables in logistic regression. Source Code Biol. Med. 3:17. 10.1186/1751-0473-3-17 19087314 PMC2633005

[B5] Chang K. V. Hsu T. H. Wu W. T. Huang K. C. Han D. S. (2016). Association between sarcopenia and cognitive impairment: a systematic review and meta-analysis. J. Am. Med. Dir. Assoc. 17, 1164.e7–e15. 10.1016/j.jamda.2016.09.013 27816484

[B6] Cho Y. J. Kim Y. J. Kim M. Y. Shin Y. J. Lee J. Choi E.. (2020). Validation of the good outcome following attempted resuscitation (GO-FAR) score in an East Asian population. Resuscitation 150, 36–40. 10.1016/j.resuscitation.2020.02.035 32194163

[B7] Cruz-Jentoft A. J. Bahat G. Bauer J. Boirie Y. Bruyère O. Cederholm T.. (2019). Sarcopenia: revised European consensus on definition and diagnosis. Age Ageing 48, 16–31. 10.1093/ageing/afy169 30312372 PMC6322506

[B8] De Buyser S. L. Petrovic M. Taes Y. E. Toye K. R. Kaufman J. M. Lapauw B.. (2016). Validation of the FNIH sarcopenia criteria and SOF frailty index as predictors of long-term mortality in ambulatory older men. Age Ageing 45, 602–608. 10.1093/ageing/afw071 27126327

[B9] Drescher C. Konishi M. Ebner N. Springer J. (2015). Loss of muscle mass: current developments in cachexia and sarcopenia focused on biomarkers and treatment. J. Cachexia Sarcopenia Muscle 6, 303–311. 10.1002/jcsm.12082 26676067 PMC4670737

[B10] Ebell M. H. Afonso A. M. (2011). Pre-arrest predictors of failure to survive after in-hospital cardiopulmonary resuscitation: a meta-analysis. Fam. Pract. 28, 505–515. 10.1093/fampra/cmr023 21596693

[B11] George N. Thai T. N. Chan P. S. Ebell M. H. (2020). Predicting the probability of survival with mild or moderate neurological dysfunction after in-hospital cardiopulmonary arrest: the GO-FAR 2 score. Resuscitation 146, 162–169. 10.1016/j.resuscitation.2019.12.001 31821836

[B12] Girotra S. Nallamothu B. K. Spertus J. A. Li Y. Krumholz H. M. Chan P. S. (2012). Trends in survival after in-hospital cardiac arrest. N. Engl. J. Med. 367, 1912–1920. 10.1056/NEJMoa1109148 23150959 PMC3517894

[B13] Huh J. W. Lim C. M. Koh Y. Lee J. Jung Y. K. Seo H. S.. (2014). Activation of a medical emergency team using an electronic medical recording-based screening system. Crit. Care Med. 42, 801–808. 10.1097/CCM.0000000000000031 24335439

[B14] Kamo N. Kaido T. Hamaguchi Y. Okumura S. Kobayashi A. Shirai H.. (2019). Impact of sarcopenic obesity on outcomes in patients undergoing living donor liver transplantation. Clin. Nutr. 38, 2202–2209. 10.1016/j.clnu.2018.09.019 30482562

[B15] Kang J. Y. Kim Y. J. Shin Y. J. Huh J. W. Hong S. B. Kim W. Y. (2019). Association between time to defibrillation and neurologic outcome in patients with in-hospital cardiac arrest. Am. J. Med. Sci. 358, 143–148. 10.1016/j.amjms.2019.05.003 31200920

[B16] Kazaure H. S. Roman S. A. Sosa J. A. (2013). Epidemiology and outcomes of in-hospital cardiopulmonary resuscitation in the United States, 2000-2009. Resuscitation 84, 1255–1260. 10.1016/j.resuscitation.2013.02.021 23470471

[B17] Levin D. C. Parker L. Rao V. M. (2017). Recent trends in imaging use in hospital settings: implications for future planning. J. Am. Coll. Radiol. 14, 331–336. 10.1016/j.jacr.2016.08.025 27884633

[B18] Martin L. Birdsell L. Macdonald N. Reiman T. Clandinin M. T. McCargar L. J.. (2013). Cancer cachexia in the age of obesity: skeletal muscle depletion is a powerful prognostic factor, independent of body mass index. J. Clin. Oncol. 31, 1539–1547. 10.1200/JCO.2012.45.2722 23530101

[B19] Marzetti E. Calvani R. Tosato M. Cesari M. Di Bari M. Cherubini A.. (2017). Sarcopenia: an overview. Aging Clin. Exp. Res. 29, 11–17. 10.1007/s40520-016-0704-5 28155183

[B20] Moisey L. L. Mourtzakis M. Cotton B. A. Premji T. Heyland D. K. Wade C. E.. (2013). Skeletal muscle predicts ventilator-free days, ICU-free days, and mortality in elderly ICU patients. Crit. Care 17:R206. 10.1186/cc12901 24050662 PMC4055977

[B21] Montano-Loza A. J. Angulo P. Meza-Junco J. Prado C. M. M. Sawyer M. B. Beaumont C.. (2016). Sarcopenic obesity and myosteatosis are associated with higher mortality in patients with cirrhosis. J. Cachexia Sarcopenia Muscle 7, 126–135. 10.1002/jcsm.12039 27493866 PMC4864157

[B22] Mourtzakis M. Prado C. M. Lieffers J. R. Reiman T. McCargar L. J. Baracos V. E. (2008). A practical and precise approach to quantification of body composition in cancer patients using computed tomography images acquired during routine care. Appl. Physiol. Nutr. Metab. 33, 997–1006. 10.1139/H08-075 18923576

[B23] Ohlsson M. A. Kennedy L. M. Ebell M. H. Juhlin T. Melander O. (2016). Validation of the good outcome following attempted resuscitation score on in-hospital cardiac arrest in southern Sweden. Int. J. Cardiol. 221, 294–297. 10.1016/j.ijcard.2016.06.146 27404694

[B24] Park H. J. Shin Y. Park J. Kim H. Lee I. S. Seo D. W.. (2020). Development and validation of a deep learning system for segmentation of abdominal muscle and fat on computed tomography. Korean J. Radiol. 21, 88–100. 10.3348/kjr.2019.0470 31920032 PMC6960305

[B25] Pedersen B. K. (2009). The diseasome of physical inactivity–and the role of myokines in muscle–fat cross talk. J. Physiol. 587, 5559–5568. 10.1113/jphysiol.2009.179515 19752112 PMC2805368

[B26] Polyzos S. A. Margioris A. N. (2018). Sarcopenic obesity. Hormones 17, 321–331. 10.1007/s42000-018-0049-x 30014320

[B27] Rittenberger J. C. Raina K. Holm M. B. Kim Y. J. Callaway C. W. (2011). Association between cerebral performance category, modified rankin scale, and discharge disposition after cardiac arrest. Resuscitation 82, 1036–1040. 10.1016/j.resuscitation.2011.03.034 21524837 PMC3138855

[B28] Steffl M. Bohannon R. W. Sontakova L. Tufano J. J. Shiells K. Holmerova I. (2017). Relationship between sarcopenia and physical activity in older people: a systematic review and meta-analysis. Clin. Interv. Aging 12, 835–845. 10.2147/CIA.S132940 28553092 PMC5441519

[B29] Walsh K. (2009). Adipokines, myokines and cardiovascular disease. Circ. J. 73, 13–18. 10.1253/circj.CJ-08-0961 19043226

[B30] Wang C. H. Chang W. T. Huang C. H. Tsai M. S. Yu P. H. Wu Y. W.. (2018). Validation of the cardiac arrest survival postresuscitation in-hospital (CASPRI) score in an East Asian population. PLoS ONE 13:e0202938. 10.1371/journal.pone.0202938 30138383 PMC6107241

[B31] Wells J. M. Washko G. R. Han M. K. Abbas N. Nath H. Mamary A. J.. (2012). Pulmonary arterial enlargement and acute exacerbations of COPD. N. Engl. J. Med. 367, 913–921. 10.1056/NEJMoa1203830 22938715 PMC3690810

[B32] WHO Expert Consultation (2004). Appropriate body-mass index for Asian populations and its implications for policy and intervention strategies. Lancet 363, 157–63. 10.1016/S0140-6736(03)15268-3 14726171

[B33] Wong A. Frishman W. (2019). Sarcopenia and cardiac dysfunction. Cardiol. Rev. 28, 197–202. 10.1097/CRD.0000000000000285 31868771

[B34] Yoon J. C. Kim Y. J. Lee Y. J. Ryoo S. M. Sohn C. H. Seo D. W.. (2018). Serial evaluation of SOFA and APACHE II scores to predict neurologic outcomes of out-of-hospital cardiac arrest survivors with targeted temperature management. PLoS ONE 13:e0195628. 10.1371/journal.pone.0195628 29621337 PMC5886591

[B35] Zhang X. Xie X. Dou Q. Liu C. Zhang W. Yang Y.. (2019). Association of sarcopenic obesity with the risk of all-cause mortality among adults over a broad range of different settings: a updated meta-analysis. BMC Geriatr. 19:183. 10.1186/s12877-019-1195-y 31269909 PMC6610788

