# Supplementary material for: Long-Term Outcomes After In-Hospital Cardiac Arrest: Does Pre-arrest Skeletal Muscle Depletion Matter?
Source: Front Physiol. 2021 Jul 29;12:692757. doi: 10.3389/fphys.2021.692757 (PMC8359293; doi:10.3389/fphys.2021.692757)
Supplement: Supplementary file 1 [file Table_1.DOCX]

Supplementary Material

Supplementary Table S1. Univariable logistic analysis for good neurological outcome at 6 months

| **Variables** | **Good neurological outcome at 6 months** | |
| --- | --- | --- |
|  | **Crude OR (95% CI)** | ***p*** |
| **Demographics** |  |  |
| Age | 1.002 (0.983–1.022) | 0.837 |
| Male | 0.784 (0.457–1.346) | 0.378 |
| **Comorbidities** |  |  |
| Hypertension | 0.981 (0.565–1.703) | 0.946 |
| Diabetes mellitus | 0.553 (0.294–1.041) | 0.066* |
| Coronary artery disease | 1.273 (0.623–2.602) | 0.508 |
| Heart failure | 1.382 (0.655–2.918) | 0.396 |
| Chronic pulmonary disease | 0.944 (0.316–2.818) | 0.917 |
| Chronic kidney disease | 0.766 (0.360–1.630) | 0.489 |
| Liver cirrhosis | 0.714 (0.291–1.750) | 0.461 |
| Active cancer | 0.449 (0.258–0.781) | 0.005* |
| **Characteristics of arrest** |  |  |
| Witnessed | 10.065 (1.365–74.235) | 0.024* |
| Shockable rhythm | 2.933 (1.594–5.399) | 0.001* |
| Resuscitation duration (min) | 0.906 (0.870–0.944) | < 0.001* |
| Presumed cardiac cause | 2.719 (1.510–4.898) | 0.001* |
| CASPRI score | 0.775 (0.727–0.826) | < 0.001 |
| GO-FAR score | 0.885 (0.849–0.922) | < 0.001 |
| **Absence of skeletal muscle depletion** | 2.151 (1.250–3.700) | 0.006* |

CASPRI, cardiac arrest survival post-resuscitation in-hospital, CI, confidence interval; CPC, cerebral performance score; GO-FAR, good outcome following attempted resuscitation OR, odds ratio

* Variables that had p-values of <0.1 in the univariable analysis were entered into a multivariable analysis. However, CASPRI and GO-FAR score were not considered in the multivariable analysis owing to the possibility of confounding effects.

Supplementary Table S2. Relationship between skeletal muscle depletion and good neurological outcome according to the presence of obesity

|  | **Skeletal muscle depletion (N=248)** | |  |
| --- | --- | --- | --- |
| **Outcomes** | **Non-obese, VFA* <100 (N=132)** | **Obese, VFA ≥100 (N=116)** | ***p*** |
| Sustained ROSC | 85 (64.4) | 83 (71.6) | 0.276 |
| **At discharge** |  |  |  |
| Survival | 34 (25.8) | 22 (19.0) | 0.225 |
| Good (CPC 1–2) | 19 (14.4) | 11(9.5) | 0.250 |
| **At 1 month** |  |  |  |
| Survival | 29 (22.0) | 21 (18.1) | 0.526 |
| Good (CPC 1–2) | 18 (13.6) | 10 (8.6) | 0.234 |
| **At 6 months** |  |  |  |
| Survival | 22 (16.7) | 16 (13.8) | 0.598 |
| Good (CPC 1–2) | 17 (12.9) | 10 (8.6) | 0.313 |

CPC, cerebral performance category; ROSC, return of spontaneous circulation; VFA, visceral fat area

Data are expressed as a number with a percentage.

* Obesity is defined as VFA of ≥100 cm^2^, and the units are omitted in the table.
